# Bioengineering of vascularized porcine flaps using perfusion-recellularization

**DOI:** 10.1038/s41598-024-58095-7

**Published:** 2024-03-31

**Authors:** Michael S. Xu, Andrew D’Elia, Nina Hadzimustafic, Aisha Adil, Golnaz Karoubi, Thomas K. Waddell, Siba Haykal

**Affiliations:** 1 Latner Thoracic Surgery Research Laboratories, University Health Network, 200 Elizabeth Street 8N-869, Toronto, ON M5G 2C4 Canada; 2 Division of Thoracic Surgery, University of Toronto, Toronto, ON Canada; 3 Plastic and Reconstructive Surgery, Smilow Cancer Hospital, Yale New Haven Health, New Haven, CT USA

**Keywords:** Mesenchymal stem cells, Biological models

## Abstract

Large volume soft tissue defects greatly impact patient quality of life and function while suitable repair options remain a challenge in reconstructive surgery. Engineered flaps could represent a clinically translatable option that may circumvent issues related to donor site morbidity and tissue availability. Herein, we describe the regeneration of vascularized porcine flaps, specifically of the omentum and tensor fascia lata (TFL) flaps, using a tissue engineering perfusion-decellularization and recellularization approach. Flaps were decellularized using a low concentration sodium dodecyl sulfate (SDS) detergent perfusion to generate an acellular scaffold with retained extracellular matrix (ECM) components while removing underlying cellular and nuclear contents. A perfusion-recellularization strategy allowed for seeding of acellular flaps with a co-culture of human umbilical vein endothelial cell (HUVEC) and mesenchymal stromal cells (MSC) onto the decellularized omentum and TFL flaps. Our recellularization technique demonstrated evidence of intravascular cell attachment, as well as markers of endothelial and mesenchymal phenotype. Altogether, our findings support the potential of using bioengineered porcine flaps as a novel, clinically-translatable strategy for future application in reconstructive surgery.

## Introduction

Large and complex soft tissue defects following cancer resection, traumatic injuries, or severe burns can cause significant impairment to patient quality of life arising from severe loss of function or permanent disability. While autologous tissue transfer using flaps is a commonly used technique in reconstructive surgery, issues with flap availability and donor site morbidity are major limitations^1–3^. Bioengineered, animal-derived, vascularized flaps present a potentially translatable “off-the-shelf option” for use in reconstructive surgery. Such an option would circumvent limitations of donor site morbidity or tissue availability associated with current conventional reconstructive surgery using free flaps. These tissues, however, present their own host of challenges—the most significant being immunogenicity.

Tissue-engineered constructs can potentially overcome the problems associated with animal-derived flaps for reconstructive surgery^4,5^. One such tissue engineering method involves the removal of cellular material from native tissue while preserving the underlying extracellular matrix (ECM), resulting in an acellular scaffold. In contrast to the use of synthetic materials in tissue engineering, acellular matrices derived from biological sources provide a higher fidelity scaffold for engineered tissues because of their physiological resemblance to native tissues, including intact microarchitecture, preserved ECM components, vascular network, and biomechanical properties^6^. Biologically derived scaffolds also contain native endogenous signals, which provide an optimal biological, biochemical and biophysical environment to guide cell survival, proliferation, fate determination, and behavior^6,7^. Recellularizing these acellular matrices with recipient-derived undifferentiated cells can functionalize the tissue while mitigating the immunogenicity of native xenografts.

Various tissue decellularization methods have been attempted by the scientific community with favourable results, including whole organ perfusion, immersion and agitation, pressure gradient, and supercritical fluids^8^. In particular, perfusion-decellularization offers a facile and effective approach for the decellularization of vascularized tissues, wherein detergents with solubilizing and/or enzymatic qualities are passed through the vascular network using a pump-based system^9^. Many studies have demonstrated the use of low-concentration SDS to be optimal in the perfusion-decellularization process, as it preserves ECM microarchitecture and is significantly less cytotoxic, permitting for recellularization^10–12^.

Recently, progress in recellularization of biological tissues has also been documented in acellular scaffolds using vascular cell populations in order to regenerate the vasculature. Within the setting of tissue regeneration, efforts utilizing decellularization and recellularization techniques have been performed in whole organs such as the kidney^13^, liver^14^, heart^15^, and lung^16,17^. Similar applications using tissue engineering have also been studied beyond solid organs in vascularized composite allografts such as rodent^18^ and primate^19^ forelimb, as well as rat and porcine fasciocutaneous flaps^20,21^, and porcine and human ear grafts^22,23^. A major challenge with recellularization is achieving adequate cell density in order to promote tissue regeneration with host cells and prevent scaffold degeneration. Considering the importance of the vasculature in supporting viable tissues during free flap reconstructive surgery, we focused on a recellularization strategy for bioengineered flaps that regenerated the internal endothelial layer of the vasculature. Being able to regenerate perfusable and functional flaps amenable to transplantation requires both a perfusable arteriovenous vascular architecture as well as an intact endothelium to cover exposed underlying collagen of the ECM and thereby prevent thrombosis.

Herein we study the feasibility and effectiveness of a perfusion-recellularization protocol for two clinically relevant free flaps: omentum and tensor fascia lata (TFL). The omentum is an extraordinarily versatile tissue which has shown utility in reconstruction of almost all anatomic regions. Specifically, omental flaps have been used clinically for wound coverage, lymphedema treatment, reconstruction of the chest wall^24^ and protection from anastomotic leaks^25,26^. Musculocutaneous flaps are of particular interest due to tensile strength^27^, and their broad applications^28,29^. We have selected the TFL flap as a representative musculocutaneous flap model for our study.

**Figure 1:**
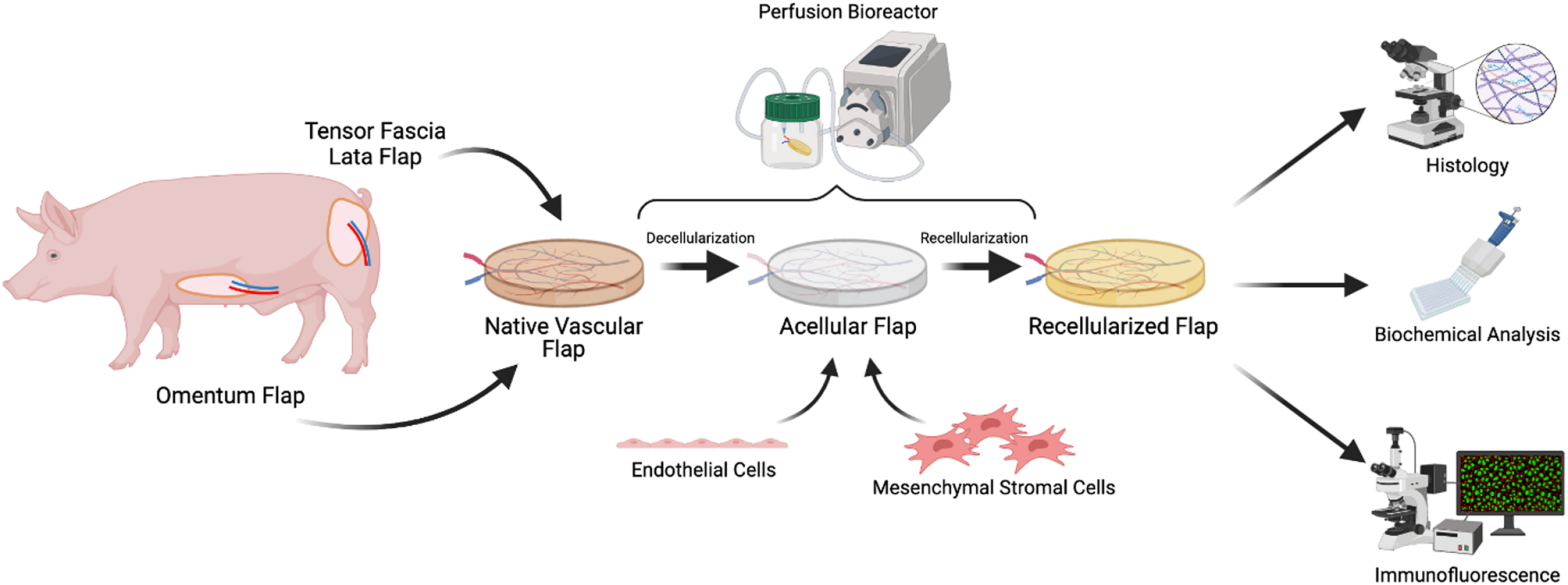
Graphical overview of tissue perfusion-decellularization and recellularization of porcine omentum and tensor fascia lata flaps. Perfusion was achieved using a multichannel controllable peristaltic pump to deliver decellularization detergent via the arterial inlet of the vascularized flap. Recellularization was achieved using a co-culture of human umbilical vein endothelial cell and human mesenchymal stem cell, manually perfused in the arterial inlet and cultured within an ex vivo perfusion bioreactor. Figure created in BioRender—biorender.com (Toronto, ON).

In this work we describe the decellularization and subsequent recellularization of porcine omental and TFL free flaps in a custom perfusion bioreactor (Fig. 1). We assess the ability of the current protocol to regenerate neo-endothelium following perfusion-culture, and describe strategies to increase recellularization efficacy. This study is thus an important development in decellularization-recellularization methods for free flaps in reconstructive surgery.

## Results

### Perfusion decellularization of porcine vascularized flaps removes nuclear material and significantly reduces DNA content

**Figure 2:**
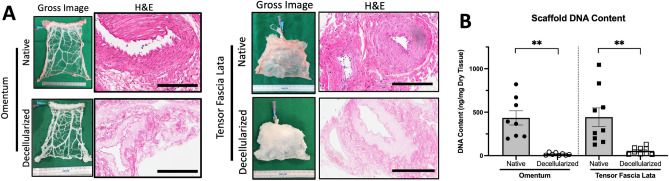
Perfusion-decellularization of vascularized porcine flaps. (**A**) After SDS perfusion-decellularization, both omentum and TFL flaps have characteristically white and opaque appearance on gross inspection in comparison to pink appearance of native tissues. Histological staining by H&E also demonstrated the absence of blue nuclear staining from hematoxylin. Representative images from n = 3 native and decellularized flaps. Scale Bars: 200 µm. (**B**) Confirmation of decellularized tissue flaps demonstrated statistically significant decrease in DNA content in decellularized tissues compared to native tissues. DNA Quantitation used Quant-iT PicoGreen dsDNA Assay against known lambda-phage DNA standard curve. Statistical testing used multiple unmatched t-tests with significance (**) level defined as p-value < 0.05; N = 8.

Vascularized porcine flaps were perfusion-decellularized through the flap vasculature using low concentration anionic SDS detergent within a customized bioreactor, based on a previously published protocol^30^. Decellularization was macroscopically evidenced by the characteristic white/opaque appearance of decellularized tissues (not pictured). Microscopically, sections stained with hematoxylin and eosin (H&E) histology showed loss of cellular material within the scaffold as indicated by the absence of blue nuclear hematoxylin staining (Fig. 2A). Confirmation of decellularization was corroborated by deoxyribonucleic acid (DNA) quantification. In the omentum, DNA content was observed to be decreased from 435 ± 82 ng/mg dry tissue in the native flap to 14.3 ± 4.2 ng/mg dry tissue in the decellularized flap (n = 8, p < 0.05). In the TFL, DNA decreased from 421 ± 98.5 ng/mg to 48.5 ± 10.3 ng/mg between native and decellularized flaps, respectively (n = 8, p < 0.05) (Fig. 2B).

### Perfusion-decellularization of porcine vascularized flaps preserves major ECM components

**Figure 3:**
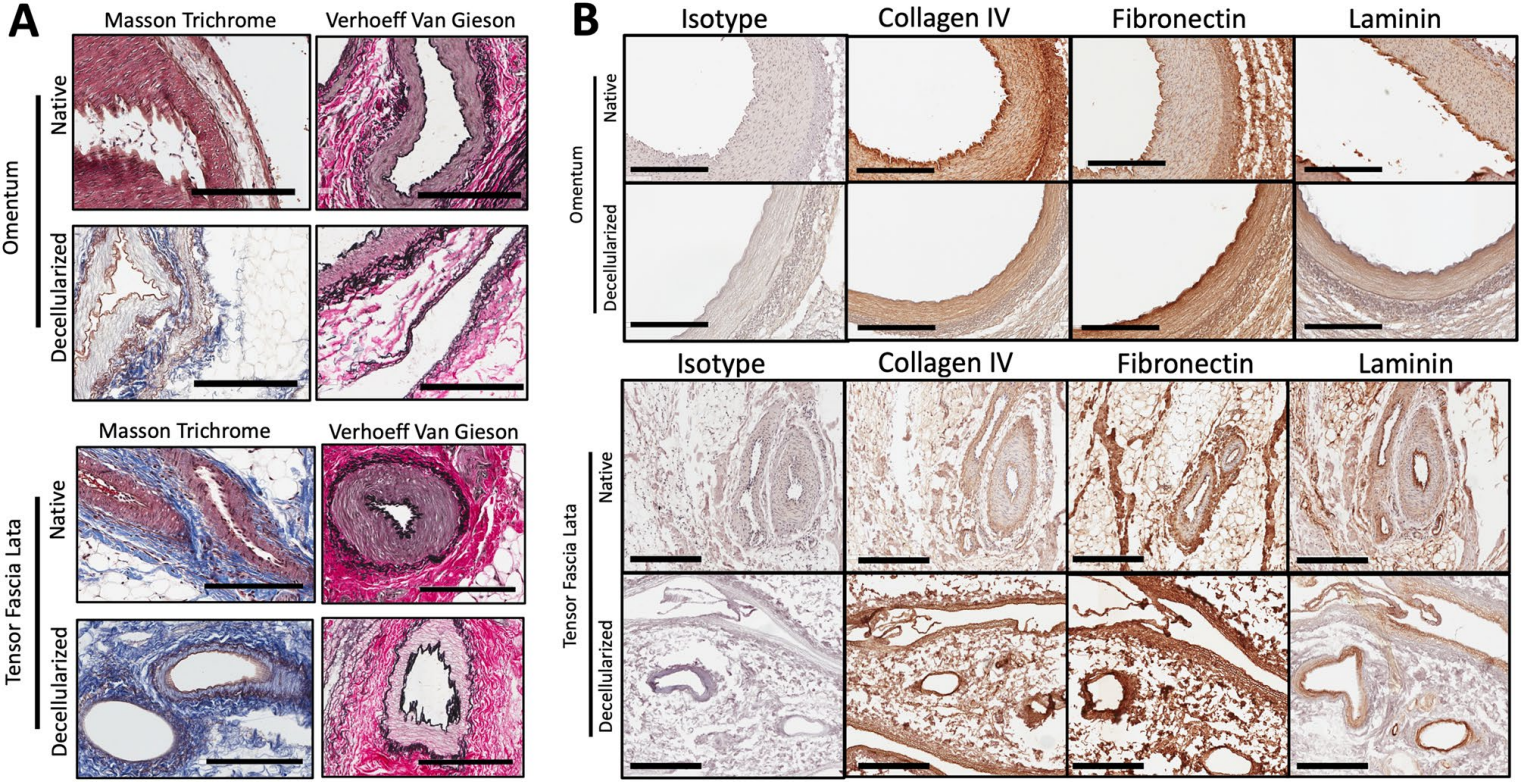
Characterization of extracellular matrix components in decellularized tensor fascia flaps. (**A**) After SDS perfusion-decellularization, histological staining using Masson Trichrome for collagen expression, and Verhoeff Van Gieson Staining for elastin expression were performed demonstrating retention in decellularized scaffolds. (**B**) Immunohistochemistry for Collagen IV, Fibronectin, and Laminin content was also performed to demonstrate their retention in decellularized scaffolds. IHC used Rabbit Ig Polyclonal Primary antibodies with Goat anti-rabbit IgG HRP-conjugated detection system. All images are representative images of n = 3 samples in both native and decellularized tissue groups. Scale Bars: 200 µm.

ECM fibrous proteins collagen and elastin were assessed histologically using Masson Trichrome and Verhoeff-Van Gieson staining, respectively. Staining demonstrated that both major ECM components were retained within the decellularized scaffolds in comparison to native tissues (Fig. 3A). Components comprising the vascular ECM, such as collagen IV, fibronectin, and laminin were also examined with immunohistochemistry (IHC). Figure 3B shows that the latter ECM components were retained in both decellularized omentum and TFL free flaps.

**Figure 4:**
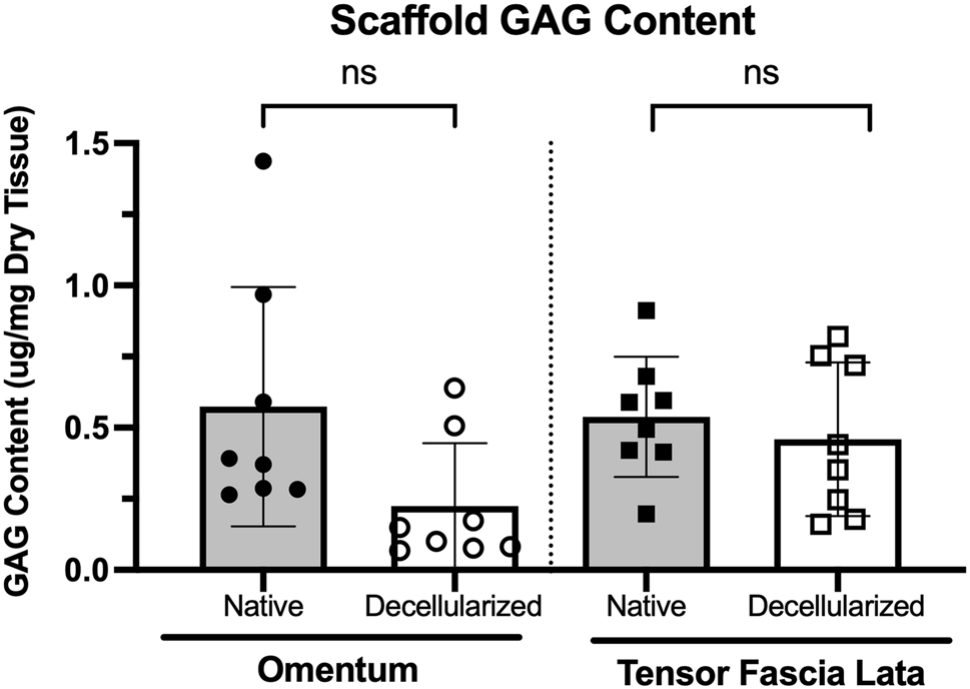
Perfusion-decellularization maintained GAG content in vascularized flaps. GAG values were normalized to milligrams of dry tissue mass. Fresh tissue samples were dried and weighed before overnight digestion in papain (0.1 mg/mL) prior to quantification. GAG assayed using Blyscan Sulfated GAG Kit. Statistical testing used multiple unmatched *t* tests with significance level defined as p-value < 0.05. *ns* not-significant. N = 8.

Glycosaminoglycan (GAG) quantification of the two decellularized flaps was also performed. In the decellularized omentum, lower GAG content was detected compared to the native omentum, however this did not reach statistical significance: 0.57 ± 0.42 µg/mg dry tissue in the native flap to 0.22 ± 0.22 µg/mg dry tissue in the decellularized flap (n = 8, p > 0.05). In the TFL, GAG content was comparable between native and decellularized condition: 0.53 ± 0.21 µg/mg to 0.46 ± 0.27 µg/mg between native and decellularized flaps, respectively (n = 8, p > 0.05) (Fig. 4).

### Decellularized porcine flaps maintain a perfusable arterio-venous vascular loop

**Figure 5:**
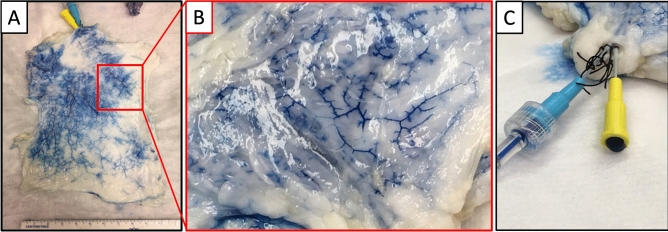
Preserved flap microcirculatory network after perfusion-decellularization. Instillation of vascular dye to visualize the microcirculatory beds of the decellularized TFL Flap was achieved by the use of Evans Blue Dye (0.05% w/v) injected into the intra-arterial cannula. Following injection, blue dye was grossly visible within the flap macrovasculature (**A**) as well as fine, distal microcirculatory vessels at closer inspection (**B**). The presence of dye outflow from venous cannula was also noted, indicating a perfusable decellularized arteriovenous loop (**C**).

Decellularized flaps were further characterized using colored Evans Blue intravascular dye. Dye was perfused through the arterial inlet of the TFL flap to opacify the underlying microvascular architecture following decellularization with SDS (Fig. 5). Fine distal microvessels in the scaffold were opacified without extravasation into surrounding parenchyma, suggesting retention of the microvascular architecture following decellularization. Additionally, blue dye was observed to flow out from the venous cannula during dye injection, thereby suggesting that a patent arterial to venous vascular circuit with connecting capillaries was intact and perfusable.

### Bioreactor set-up for perfusion-recelluarization of acellular porcine flaps

**Figure 6:**
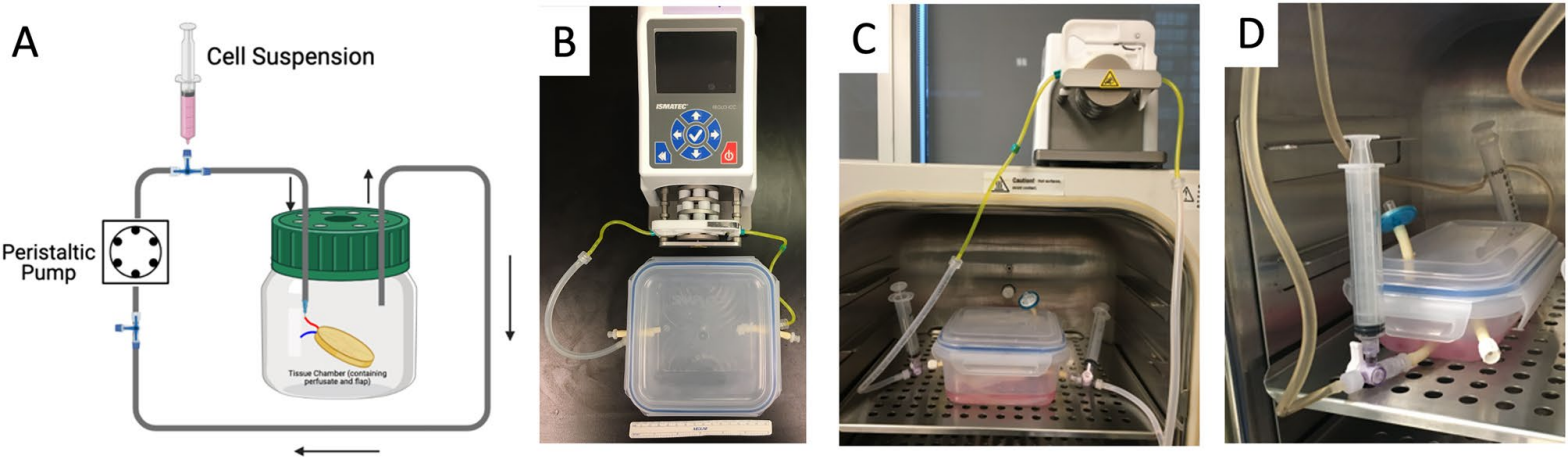
Perfusion-recellularization bioreactor system set-up. The bioreactor consisted of air-tight snap lid container with connected tubing (**A**) with unidirectional flow of culture media. The assembled bioreactor with chamber and tubing connected to peristaltic pump (**B**). The bioreactor fits within a conventional cell culture incubator with pump placed externally (**C**,**D**).

The bioreactor system for perfusion-recellularization was demonstrated in Fig. 6. This ex vivo perfusion culture system utilizes a peristaltic pump for a total duration of 6 days. Endothelial cell growth media 2 (EGM2) is used. The bioreactor was continuously monitored for contamination over the course of ex vivo perfusion culture by daily visual inspection of the bioreactor media. The main components of the recellularization-bioreactor consisted of an autoclavable, enclosed chamber to accommodate closed-loop, unidirectional perfusion of cell culture media and compatibility within a conventional tissue culture incubator at standard conditions (37 °C/5% CO_2_). A three-way stopcock proximal to the flap was used for syringe cell seeding. The peristaltic pump was mounted externally to the incubator set-up to allow for perfusion.

### Perfusion culture of HUVEC-MSCs in decellularized Scaffolds demonstrated cell engraftment to the intravascular lumen

**Figure 7:**
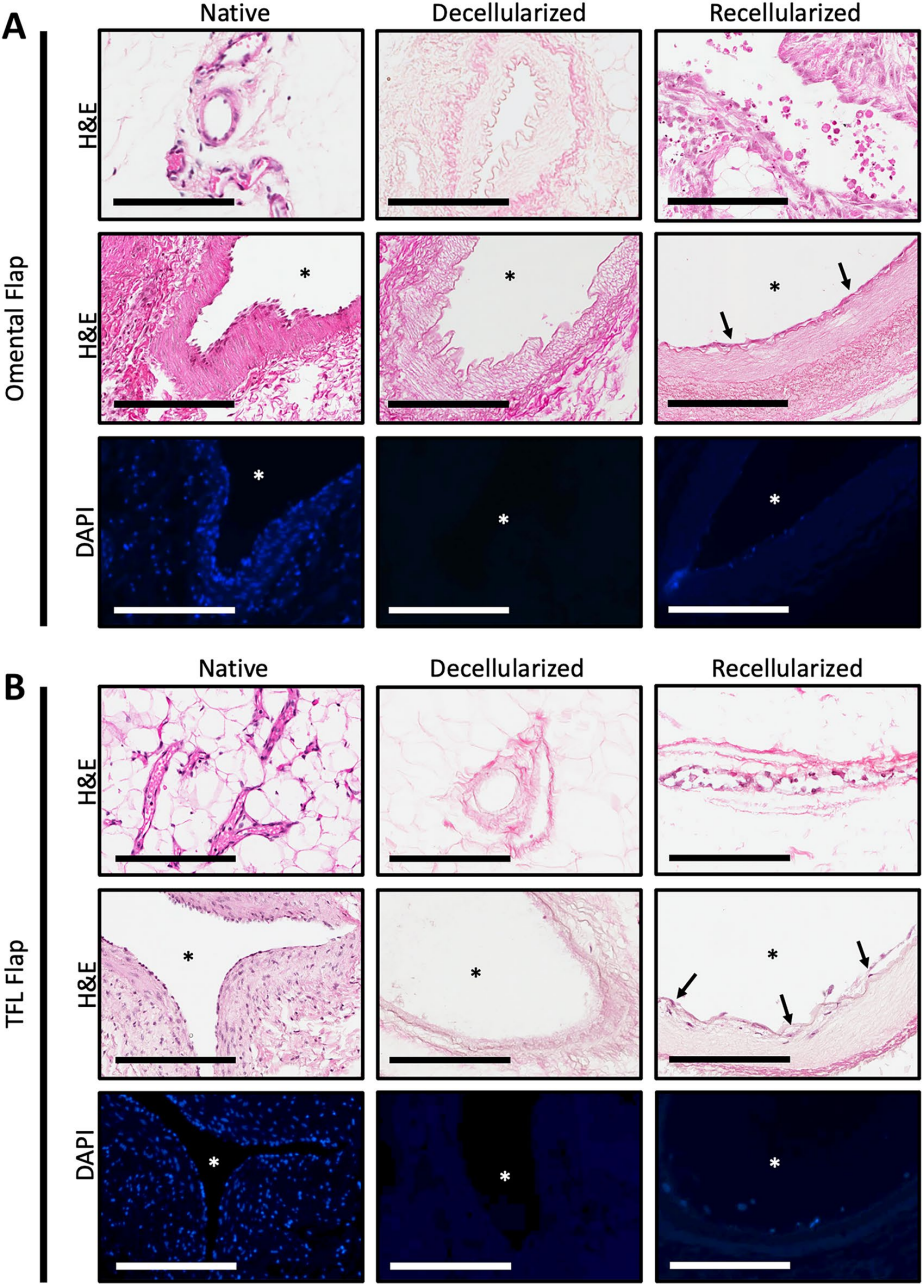
Recellularization of acellular omentum and tensor fascia lata flaps. Forty million HUVEC and forty million MSCs were manually seeded with syringe via the arterial inlet. Following 4 h of static culture, scaffolds were perfused with EGM2 growth media for a total culture period of 6 days. Evidence of cell attachment as noted in the recellularized omentum (**A**) and the tensor fascia lata (**B**) at the interface of the vascular lumen (asterisks) determined using H&E and DAPI visualization. H&E stains of small and relatively larger caliber vessels are depicted in top and middle row, respectively. Images are representative of n = 3 of each condition: native, decellularized and recellularized tissues. Scale Bar: 200 µm.

Recellularization of porcine flaps was achieved using a custom perfusion bioreactor with scaffold seeding and culture performed in an ex vivo fashion. Recellularization of the omentum and TFL flaps was performed using the two cell populations under co-culture conditions. A total of 40 × 10^6^ HUVEC with 40 × 10^6^ MSC cells were used for recellularization. In both the recellularized omentum and TFL, presence of cell engraftment to the intravascular luminal interface was seen on H&E as well as with DAPI staining after ex vivo perfusion culture after 6 days (Fig. 7). Of note, evidence of cell engraftment was found to be present intraluminally in both distal and proximal portions of the recellularized omental and TFL flaps. Although luminal coverage did demonstrate some bare areas without cell attachment. Reseeded cells were predominantly found in the arterial vasculature. Minimal cell engraftment was observed in the venous system of recellularized flaps.

### Perfusion-culture of HUVEC-MSCs in decellularized scaffolds regenerates neo-endothelium

**Figure 8:**
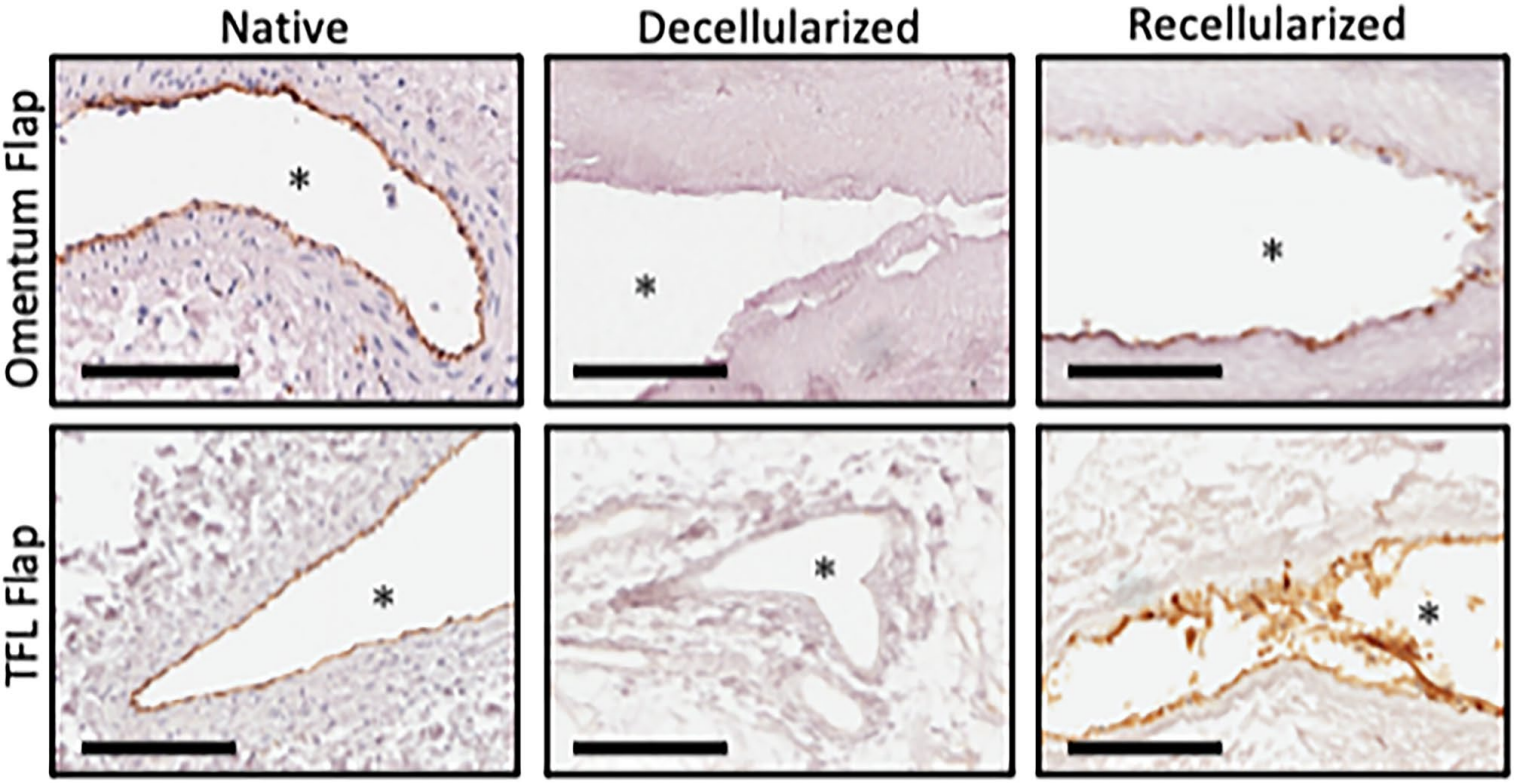
Recellularization with HUVEC and MSC co-culture regenerates endothelial phenotype in decellularized omentum and TFL flaps. Forty million HUVEC and forty million MSCs were manually seeded with syringe via the arterial inlet and cultured in bioreactor for a total of 6 days. CD31 positivity was demonstrated using IHC at the interface of the vascular lumen (asterisks) in the recellularized condition. No CD31was seen in decellularized control. Representative Images from n = 3 samples. Scale Bar: 200 µm.

We next examined the ability of the seeded endothelial cells to repopulate segments of the decellularized omentum and TFL flaps after perfusion culture. IHC analysis of flap cross-sections demonstrated a thin monolayer of cells with CD31 positivity suggesting a regenerated neo-endothelium (Fig. 8). The presence of neo-endothelium was not entirely circumferential particularly seen in the omentum, indicating some bare areas of the intravascular space.

### Recellularized flaps shows evidence of endothelial and mesenchymal phenotypes

**Figure 9:**
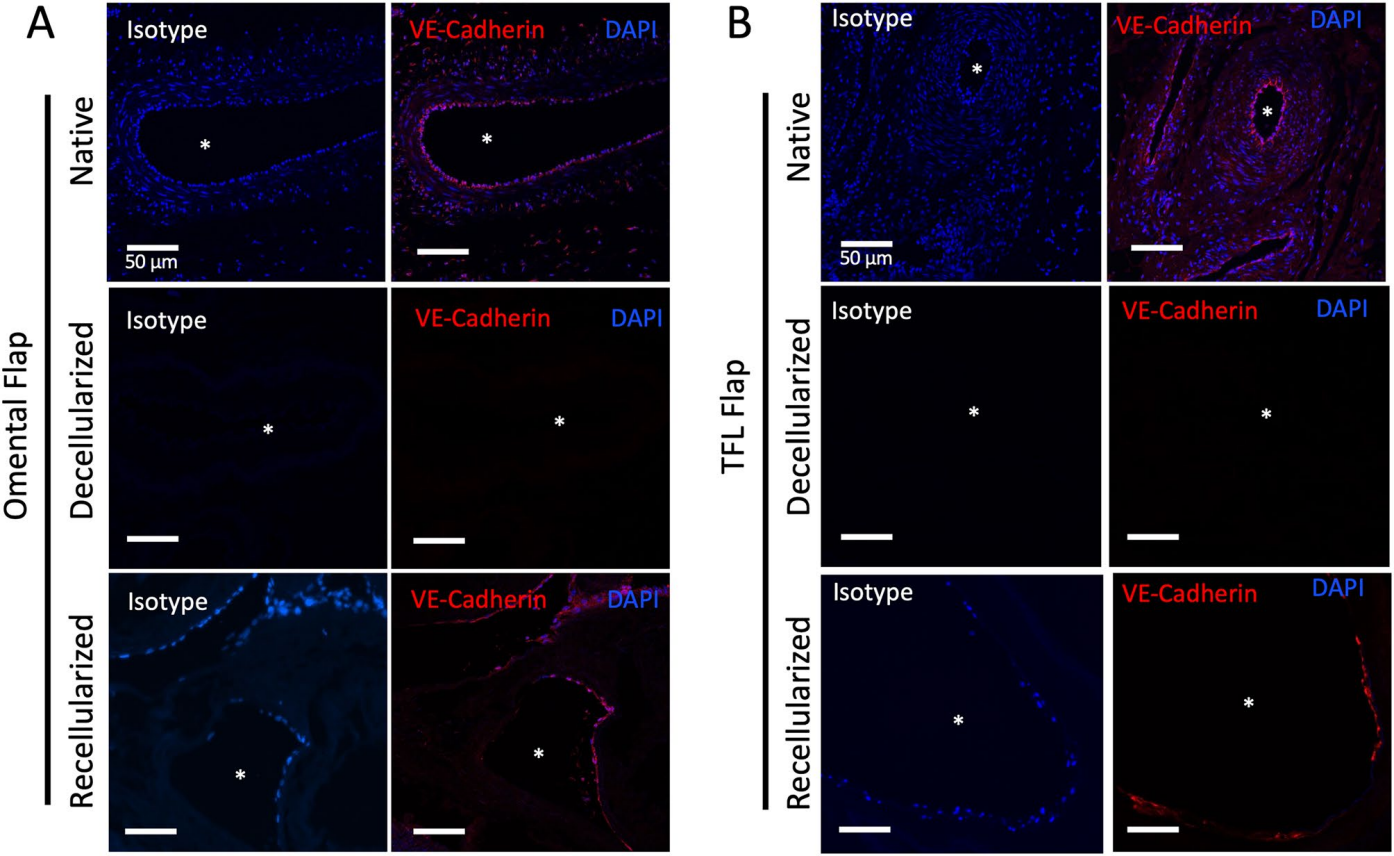
Phenotype characterization of intercellular endothelial junctional marker, VE-cadherin, after 6-day perfusion-culture. Following recellularization with HUVE -MSC co-culture for 6 days within ex vivo bioreactor, immunofluorescence demonstrates positivity for endothelial junctional marker, VE-Cadherin (red) in the recellularized omentum (**A**), and tensor fascia lata (**B**). Localization of both markers was predominant within the intravascular compartment at the luminal interface. Asterisk (*) denotes vessel lumen. Isotype control performed with rabbit IgG demonstrating negative staining. Nuclear counterstaining with DAPI (blue). Scale Bar: 50 µm.

**Figure 10:**
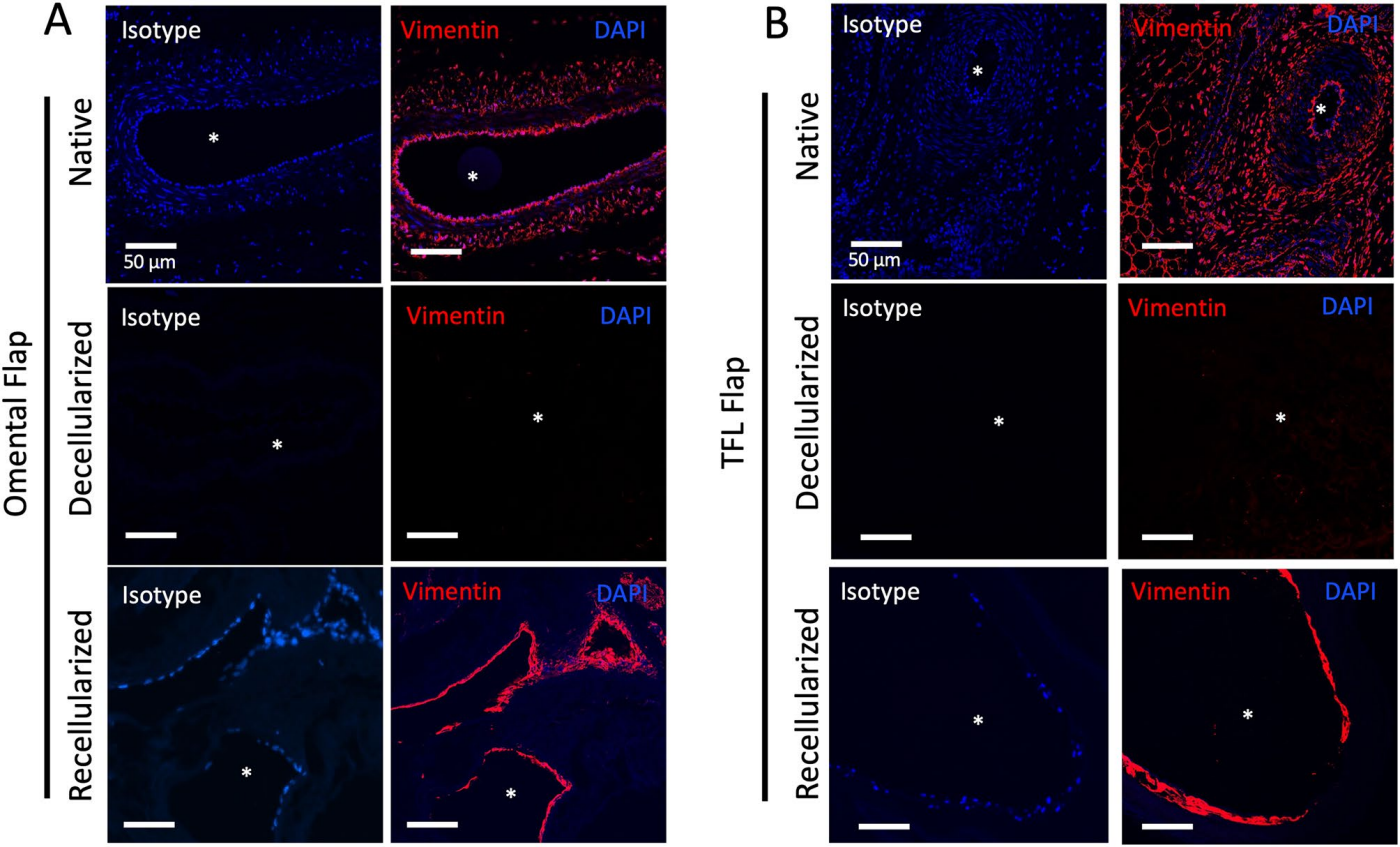
Phenotype characterization of mesenchymal marker, vimentin, after 6-day perfusion-culture. Recellularization with HUVEC-MSC co-culture for 6 days within ex vivo bioreactor, immunofluorescence demonstrated positivity for mesenchymal marker, vimentin (red) in the recellularized omentum (**A**), and tensor fascia lata (TFL) flaps (**B**). Localization of both markers was predominant within the intravascular compartment at the luminal interface. Asterisk (*) denotes vessel lumen. Isotype control performed with rabbit IgG demonstrated negative staining. Nuclear counterstaining with DAPI (blue). Scale Bar: 50 µm.

Additional phenotypic characterization of the recellularized flap was also carried out to examine the expression of the endothelial intercellular junctional marker, VE-Cadherin, as well as mesenchymal marker, vimentin. Immunofluorescence staining using VE-Cadherin and DAPI after day 6 culture showed expression of VE-Cadherin that lined the decellularized intravascular channels of both omentum and TFL flaps (Fig. 9). Expression showed the presence of a nascent monolayer of VE-Cadherin positive endothelium. However, this expression was not circumferential around the entirety of the vascular lumen, corresponding to observations seen with CD31 expression presented earlier. In the case of vimentin expression, intravascular localization of the marker was seen in the recellularized omentum and TFL flaps after 6 days in culture (Fig. 10).

## Discussion

Initial work in free flap bioengineering conducted by our lab demonstrated the feasibility and efficacy of using low-concentration SDS perfusion to generate vascularized acellular scaffolds^30^. The present work supports these results. Other groups suggest that hydrostatic pressure systems or different perfusates such as DMSO, Triton X-100, and SDC are also effective in achieving tissue decellularization including muscle^31–33^. Studies on decellularization of the greater omentum agree that perfusion with low-concentration SDS offers a superior approach to decellularization, owing to preserved microarchitecture, biochemistry, and reduced biotoxicity compared to other protocols^34^.

Although bioengineering of decellularized scaffold materials for reconstructive surgery has evolved tremendously over the last decade, efficient and effective recellularization remains challenging. It is known that effective recellularization is contingent on several factors, such as bioactivity and structural porosity, which permit and promote cell and culture media migration throughout the scaffold^31^. Scaffold biotoxicity and vascular permeability are also significant factors influencing successful recellularization^9,35^. We therefore performed a variety of tests to verify these conditions in our scaffolds prior to recellularization.

The ideal decellularization leads to an ECM which has been isolated from its native cells and genetic components without disrupting the structural integrity of the ECM. H&E staining revealed removal of cellular components between native and decellularized tissues, which in combination with DAPI staining and significant reduction of DNA content, confirmed successful decellularization of both omental and TFL free flaps. These findings are in alignment with previous reports of this decellularization protocol^30^. Next, Masson Trichrome (collagen) and Verheoff-Van Gieson (elastin) staining, immunohistochemistry for collagen IV, fibronectin, laminin, and GAG quantification assays were performed. Qualitative and quantitative findings from these methods suggest that the major components of the ECM were largely preserved. Similarly, Matuska et al. showed decellularization of the TMJ disc with optimal collagen preservation and less ECM degradation with low-concentration SDS^11^. Xing et al. noted that although high-concentration SDS is most effective in removing cellular components efficiently, it is significantly more disruptive to the ECM than low-concentration SDS^10^.

Currently absent from our analysis is quantification of the various functional growth factors that ideally remain in our scaffolds post-decellularization. This is difficult to determine accurately; contrary to our purpose, most simple protein assays measure denatured protein fragments. Lastly, microvascular perfusion highlighted by intrasvascular dye paired with observed venous return suggest that our decellularized flaps possess patent microvasculature with grossly intact architecture, which is critical for subsequent recellularization methods. This is in alignment with observed venous return in decellularized omentum and TFL previously published by our group. Haeublein et al. similarly used Evan’s blue dye to quantify vascular permeability following decellularization of the rat kidney at various SDS concentrations^36^. Although they noted an increase in vascular permeability following decellularization at both high and low SDS concentrations, the low-concentration SDS had lower loss of permeability. Other groups have reported effective decellularization, retained microarchitecture, and/or favourable biocompatibility for recellularization following conservative SDS decellularization protocols—especially in the generation of acellular cardiac prostheses^37–39^.

Next, we sought to determine if our scaffolds were also conducive to cell adhesion through perfusion-recellularization. Scaffolds were seeded with HUVECs and MSCs and cultured for 6 days under constant media-perfusion in an incubator. Although HUVECs have been used in isolation previously for re-endothelialization experiments such as Jank et al*.*^40^, studies suggest that co-culture is advantageous to monoculture conditions for purposes of cell engraftment and neovascularization. For example, MSCs have been shown to facilitate HUVEC recruitment in vivo, with pronounced blood vessel density compared to when HUVECs were grown alone^41,42^. Piard et al. note that indirect cell–cell communication and paracrine secretion when co-culturing HUVECs and MSCs have mutually beneficial effects on angiogenesis and tissue regeneration^43^. To our knowledge, this is the first study to attempt a HUVEC-MSC co-culture strategy to regenerate porcine omental and TFL flap vasculature. Notably, both HUVEC and MSCs cells used were human-derived, which offer potential clinical-translatability of our tissue engineered constructs to one day be used for in reconstructive surgery applications. Looking ahead, induced pluripotent stem cell populations may offer relatively better biocompatibility for scaffold recellularization.

The bioreactor used in our decellularization is easily modifiable for recellularization to allow for a quick and simplistic transition between steps—mitigating environmental disturbances to scaffolds and streamlining the decellularization-recellularization process. Modification of the bioreactor incorporated several features: close-circuit perfusion of cell culture media, an enclosed, autoclavable chamber for perfusion-culture under sterile conditions, and compatibility with a standard cell culture incubator environment. We anticipate future modifications to the bioreactors to incorporate real-time monitoring capabilities such as dynamic perfusion flow examinations using indocyanine green perfusion or fluorescent aided microscopy tracking to follow the course of flap re-vascularization in a relative non-invasive and non-destructive manner.

Histological investigations revealed cell engraftment within the intravascular space, suggesting adhesion and possible proliferation of seeded cells. Cell adhesion was not entirely circumferential, however, leaving portions of the vascular interface uncovered. Engraftment was also more prominent in arterial vasculature compared to the veins. CD31 and VE-Cadherin are two endothelial specific markers with important roles in vascular function: CD31 (PECAM-1) primarily mediates vascular angiogenesis, immune cell transmigration, and thrombogenesis whereas VE-Cadherin functions as an adhesive between mature endothelial cells with well-formed adherens junctions, mediating cell to cell contacts to maintain endothelial integrity and facilitating cellular communication during angio- and vasculogenesis^44^. In addition, along with previous works which highlight the safety of low concentration SDS perfusion, the presence of cells 6 days following seeding also suggests that our scaffolds did not contain cytotoxic remnants from the decellularization process, as they would otherwise be incapable of supporting cell adhesion.

Incomplete engraftment and coverage of the free flap vasculature presents opportunities for further research and optimization in scaffold recellularization. We did not observe recellularization of interstitial tissue which is a future direction in perfusion-recellularization research to achieve whole-tissue recellularization of free flaps. Various seeding protocols have become the subject of several whole organ recellularization protocols. Martinello et al. describe two approaches to tendon recellularization, wherein cells are either injected directly into the scaffold and allowed to settle, or the scaffold is pre-treated with a collagen-rich gel prior to cell injection^45^. Other recellularization methods should be explored, such as serial, retrograde/bi-directional, or higher-density seeding. Wang et al. describe seeding strategies in the context of liver recellularization, reporting trials with continuous perfusion seeding, serial/sequential seeding, and seeding with different cell types^46^. Notably, Wang et al. suggest that seeding with non-parenchymal cells can help facilitate the engraftment and positioning of other cells^46^. In free flaps, recellularization from both arterial and venous conduits could lead to more homogenous cell distribution and significantly higher coverage within the scaffold.

In order to achieve complete vascular coverage, it is also likely that more cells are needed for seeding. This highlights a significant limitation of recellularization-based methods, as large-scale expansion of primary cell populations poses a challenge to feasibility of scaled scaffold production. On the other hand, the current study also lays the groundwork for other scaffold-based reconstructive strategies: partial recellularization ex vivo (e.g. endothelialization without parenchymal seeding) followed by additional maturation in vivo using the recipient as an “in vivo bioreactor” to complete recellularization could be a potential strategy for certain reconstructive applications. Longer incubation times will also be required for generation of a more extensive vascular endothelium that resembles that of native tissues. An extended culture period would also allow assessment of the longer-term neo-angiogenesis and vascular remodeling events that occur during the recellularization process.

In summary, this proof-of-concept study describes a method allowing for recellularization and perfusion-culture of two porcine flap scaffolds within a bioreactor. The main application of this work is to permit subsequent recellularization of these acellular scaffolds with cell populations that can regenerate the vasculature. Future research is required to determine the appropriate cell numbers, populations, seeding strategies, and bioreactor conditions needed to regenerate functional and viable vascularized tissue. Additionally, the seeding method used within our bioreactor was able to achieve early adherence of vascular endothelial cells after a short duration culture. The presented study lays the groundwork for perfusion-bioreactor decellularization-recellularization strategies in soft tissue engineering for reconstructive surgery.

## Methods

### Animal use

Yorkshire pigs (30–40 kg; age approximately 12 weeks old) were used for all decellularization and recellularization experiments. All studies were approved by the Institutional Animal Care and Use Committee (IACUC) of the University Health Network and Toronto General Hospital Research Institute. Humane care was provided to all animals in accordance to the “Principles of Laboratory Animal Care” defined by the National Society for Medical Research and the “Guide for the Care of Laboratory Animals” issued by the National Institutes of Health. Reporting of use of experimental animals in this study followed recommendations specified by the ARRIVE guidelines.

### Surgical procurement of porcine omentum and tensor fascia lata flap

Pigs were fasted for 12 h prior to surgery. Sedation was achieved with ketamine (20 mg/kg IM), atropine (0.04 mg/kg IM) and midazolam (0.3 mg/kg IM). Anesthesia was induced by inhalation of 5% isoflurane through a mask at a flow rate of 22 to 44 mL/kg/min to facilitate peripheral line insertion and intubation. Anesthesia was maintained with isoflurane (0.5 to 2%). Pigs were intubated with an appropriate endotracheal tube (7–8 mm) and ventilated to a tidal volume of 8 mL/kg, positive end-expiratory pressure of 5 cm H_2_O, FiO_2_ of 0.5 and respiratory rate of 14 breaths per minute. Pigs were prepped and draped in the usual sterile fashion prior to flap procurement. Surgical procedure for porcine omentum and TFL flaps procurement were as previously described^30^. Briefly, the omental flap was procured by midline laparotomy and the left gastroepiploic artery and vein was used as the dominant vascular conduit. The right gastroepiploic vessels were ligated to prevent perfusion flow-through.

The TFL flap was procured with pigs in the lateral decubitus position. The main vascular pedicle was defined by the ascending branch of the lateral circumflex femoral artery and veins. The overlying skin island was removed to produce a pure fascial flap. Following flap detachment, the vascular pedicle was cannulated with 20–22 G Angiocath (Becton Dickenson) under direct vision and flushed with 20 U/mL heparin sodium (LEO Pharma, Denmark) in 0.9% normal saline and transported under sterile conditions to the lab.

### Perfusion decellularization of porcine vascularized flaps

Porcine flaps were perfusion-decellularized using low-concentration SDS followed by DNase (Sigma Aldrich) reconstituted to a concentration of 10 mg/mL, as previously described^30^. Cannulated flaps were each connected to a perfusion system to allow antegrade perfusion via the arterial inlet at 2 ml/min, in which solutions: 0.05% SDS followed by 0.1 mg/mL deoxyribonuclease (DNase) were perfused through the flap vasculature with 1 × phosphate buffered saline (PBS) perfusion in between to remove residual detergent. Flaps were sterilized in 0.1% paracetic acid (PAA) / 4% ethanol (EtOH) (Sigma Aldrich) and then washed in 1 × PBS prior to recellularization. As described previously^30^, omental and TFL flaps were perfused with SDS for 2 and 3 days, respectively. Following SDS perfusion, flaps were washed with PBS for 24 h and then perfused with DNase for 2 h, PBS for again for 24 h, and finally PAA/EtOH for 3 h. With the exception of DNase, each step included an exchange of the submersion fluid to match the given perfusate. For the DNase step, flaps were submerged in fresh PBS.

### In vitro culture and expansion of HUVECs and MSCs

Commercially available HUVECs (American Type Culture Collection/ATCC, USA) were cultured in EGM-2 (Lonza, Switzerland) supplemented with SingleQuots (Lonza) of Growth Supplements including: FBS 2%, hEGF, hydrocortisone, Gentamicin/Amphotericin-B, VEGF, hFGF-B, R3-IGF-1, ascorbic acid, and heparin (concentrations proprietary). Commercially obtained human bone-marrow derived MSCs (Promocell, Germany) were cultured in MSCGM (Promocell) containing proprietary media supplement and 5% FBS. HMSCs and HUVECs between passage 4 and 6 were used for recellularization. Both cell types were verified for correct functional and phenotype expression. HUVECs expressed CD31/VE-Cadherin using flow cytometry and were functionally capable to undergo angiogenesis. MSCs were CD90/73/44 positive and CD34/45/11b negative using flow cytometry and capable of undergoing trilineage differentiation (Supplementary Fig. 1). These findings were consistent with the minimal criteria to define MSCs according to the International Society for Cellular Therapy Criteria^47^.

All cells were maintained in 150 cm^2^ dishes until reaching 90% confluency (resulting in approximately 50,000 cells/cm^2^). Cells were detached from culture vessels with 0.25% trypsin–EDTA solution (Gibco) prior to recellularization. Cell media was replaced every other day, and the cultures were maintained in a humidified 95% air/5% CO_2_ incubator at 37 °C.

### Perfusion recellularization bioreactor and culture

A closed-system bioreactor was set up in an incubator for recellularization within the flap scaffold matrix. We used a modified airtight snap-lid container, previously used for decellularization with a closed-circuit L/S-16 (Masterflex, Fisher Scientific) silicone tubing. The end of the tubing external to the tissue chamber was fitted with a female Luer thread-style panel (Cole-Parmer), which connected to a 3-stop tubing compatible with peristaltic pump (Ismatec, Cole-Parmer) tubing cassette as previously used for perfusion-decellularization. The opposite end of tubing was reconnected to the second port from the tissue chamber to allow closed-loop circulation of medium from tissue chamber into the flap via the arterial cannula at a flow rate of 2 mL/min. Just proximal to the tissue chamber, silicone tubing was connected to a three-way stopcock (Baxter, USA). The chamber was filled with 200 mL of EGM-2 media, which was primed through the tubing to remove air bubbles. Decellularized flaps were perfused with EGM-2 at 2 mL/min in conventional cell culture incubator at standard conditions (95% air/5% CO_2_) overnight before cell seeding to equilibrate flaps with culture medium.

### Flap vascular seeding of HUVEC and MSC co-culture

Cell seeding was performed as follows: HUVECs and human bone-marrow derived MSCs were lifted from tissue culture plastic with 0.25% trypsin and centrifuged at 500×*g* for 5 min. The resultant cell pellet was resuspended in 10 mL media, strained with 75 µm pore mesh, and counted via automated hemocytometer (Vi-Cell XR, Beckman Coulter). A total of 8 × 10^7^ cells, divided equally with 4 × 10^7^ HUVEC co-cultured with 4 × 10^7^ MSCs, were used for recellularization of each scaffold. A combined cell suspension of the two cells were slowly manually injected into the vascular arterial inlet through a three-way stopcock. Following the introduction of cells, flaps were placed in a standard cell culture incubator for 2 h of static culture to allow cell attachment. Afterwards, perfusion-culture was initiated with the peristaltic pump (Ismatec, Cole-Parmer) running at 2 mL/min for 6 days. Media passed through the flap was recovered back into the reservoir using a separate pump channel that drained the bioreactor at an equal rate to the perfusion, allowing for recycling and reuse. Media was exchanged every other day for fresh EGM-2. A total of 750 mL of culture medium was used over 6 days for each flap.

### Histology and immunohistochemistry

Native, decellularized, and recellularized tissues were biopsied near the distal margin of the flap, fixed in 10% formalin (Fisher Scientific), embedded in paraffin, and sliced into 5 µm sections on microtome (Leica Biosystems). Slides of the paraffin-embedded samples were processed for histological and IHC staining. Histologic staining was performed on xylene-deparaffinized slides with the following stains: H&E (Sigma Aldrich), Masson’s Trichrome (American MasterTech Scientific), and Verhoeff Van Gieson Elastin Stain (Abcam).

For IHC, heat induced antigen retrieval was done with citrate buffer (pH 6.0; Thermo Fisher Scientific) in a 95 °C autoclave for 10 min. Endogenous peroxidases were blocked with a peroxide block (Cardinal Health), and nonspecific binding was blocked with Dako Serum-Free Protein-Block (Agilent). Sections were incubated with the primary antibodies at 4 °C overnight with dilutions as follows: rabbit polyclonal anti-Collagen IV (Abcam, ab6586, 1:300), rabbit polyclonal anti-Fibronectin (Abcam, ab23751; 1:400); and rabbit polyclonal anti-Laminin (Abcam, ab11575, 1:400) and anti-CD31 (Abcam, ab28364, 1:50) at 4 °C overnight. Slides were washed three times in PBS with 0.1% Tween and goat anti rabbit IgG HRP-conjugated secondary antibody (ImmPRESS Peroxidase Polymer Reagent, Vector Laboratories) was applied for 30 min. Slides were again washed thrice in PBS-Tween and then diaminobenzidine solution (Vector Laboratories) applied for 10 min. Slides were counterstained with hematoxylin. After staining, all slides were dehydrated in ethanol to xylene exchange, mounted and imaged on Aperio CS2 Slide Scanner (Leica Biosystems).

Immunofluorescence staining was performed using paraffin embedded sections cut to 5 μm thickness and deparaffinized using xylene and rehydrated in serial dilutions of ethanol. Tissue sections in were incubated in antigen retrieval buffer (10 mM citrate buffer, pH 6.0) at 95 °C for 10 min in an autoclave. Tissue sections were then blocked with 5% blocking serum (goat serum) in 1% bovine serum albumin (BSA) before adding primary antibody. Slides were then incubated with primary antibodies for VE-Cadherin (Abcam, ab33168, 1:100) and vimentin (Abcam, ab92547, dilution 1:200) diluted in 1% BSA at 4 °C overnight. After washing three times with PBS-Tween, slides were then incubated for 1 h at RT in the secondary antibody goat anti-rabbit IgG conjugated with AlexaFluor 647 (Thermo Fisher Scientific, 1:500). Finally, slides were washed three times with PBS-Tween in the dark and counterstained with DAPI (Abcam; 1:5000). Negative controls were used by replacing the primary antibody with the corresponding isotype (IgG) of the primary antibody. Images were taken on a Leica SP8 confocal microscope with LAS X software (Leica Biosystems) installed.

### GAG quantification

Tissue pieces (~ 30–40 mg) were obtained by punch biopsy tool and dried in 60 °C oven overnight. Dried tissue pieces were digested in papain solution at 65 °C for 18 h. Corresponding native flap tissues were dried and digested in parallel as controls. Papain (Sigma Aldrich,  ≥ 16 units/mg protein) 15–30 mg/mL stock was solubilized to working concentration of 0.1 mg/ml in 0.1 M phosphate buffer (pH 6.0), with 5 mM cysteine hydrochloride (Sigma Aldrich), and 5 mM EDTA (Sigma Aldrich). The lysates were used for detection of sulfated glycosaminoglycan (sGAG) and DNA content. The Blyscan Sulfated GAG Assay kit (Biocolor) was used to measure sGAG according to manufacturer’s instruction. Briefly, tissue specimen lysates were mixed with Blyscan Dye Reagent to bind the GAG for 1 h at room temperature. The GAG-dye complex was then collected by centrifugation at 10,000×*g*. After the supernatant was removed and the tube drained, Dissociation Reagent was added and 100 μl of analyte solution was transferred to a 96-well plate. Absorbance against the background control was obtained at a wavelength of 656 nm with a SpectraMax spectrophotometer (Molecular Devices). GAG amount was interpolated from a standard curve (0–5 µg) using a known GAG standard provided in the kit. Final GAG content was standardized to the total dry tissue mass (mg) used for assay.

### DNA quantification

For DNA content quantitation, the tissue lysate following papain digestion (above) was used. The Quant-iT PicoGreen dsDNA Assay Kit (Invitrogen) was used to measure DNA content according to manufacturer’s instruction. Fluorescence reading (excitation: 485 nm and emission: 528 nm) was taken on a plate reader (Cytation 5, Biotek), and the absolute amount of DNA (ng) was quantified against a lambda DNA standard curve (0–1000 ng) provided by the manufacturer; final DNA content was standardized to total dry tissue mass (mg) used for assay.

### Statistical analysis

All statistical analysis was performed using GraphPad Prism, version 9.0 (GraphPad, Inc.). Statistical analyses was conducted with multiple unpaired *t* test with a significance level of p < 0.05. Values are presented as mean, with S.D. unless stated otherwise.

### Supplementary Information

Supplementary Figure 1.

Supplementary Information.

## Data Availability

The datasets generated and/or analyzed during the current study are available in the following repository 10.6084/m9.figshare.22791206.v1.
