# Supplementary figures and images for: Bioengineering of vascularized porcine flaps using perfusion-recellularization
Source: Sci Rep. 2024 Mar 31;14:7590. doi: 10.1038/s41598-024-58095-7 (PMC10981729; doi:10.1038/s41598-024-58095-7)

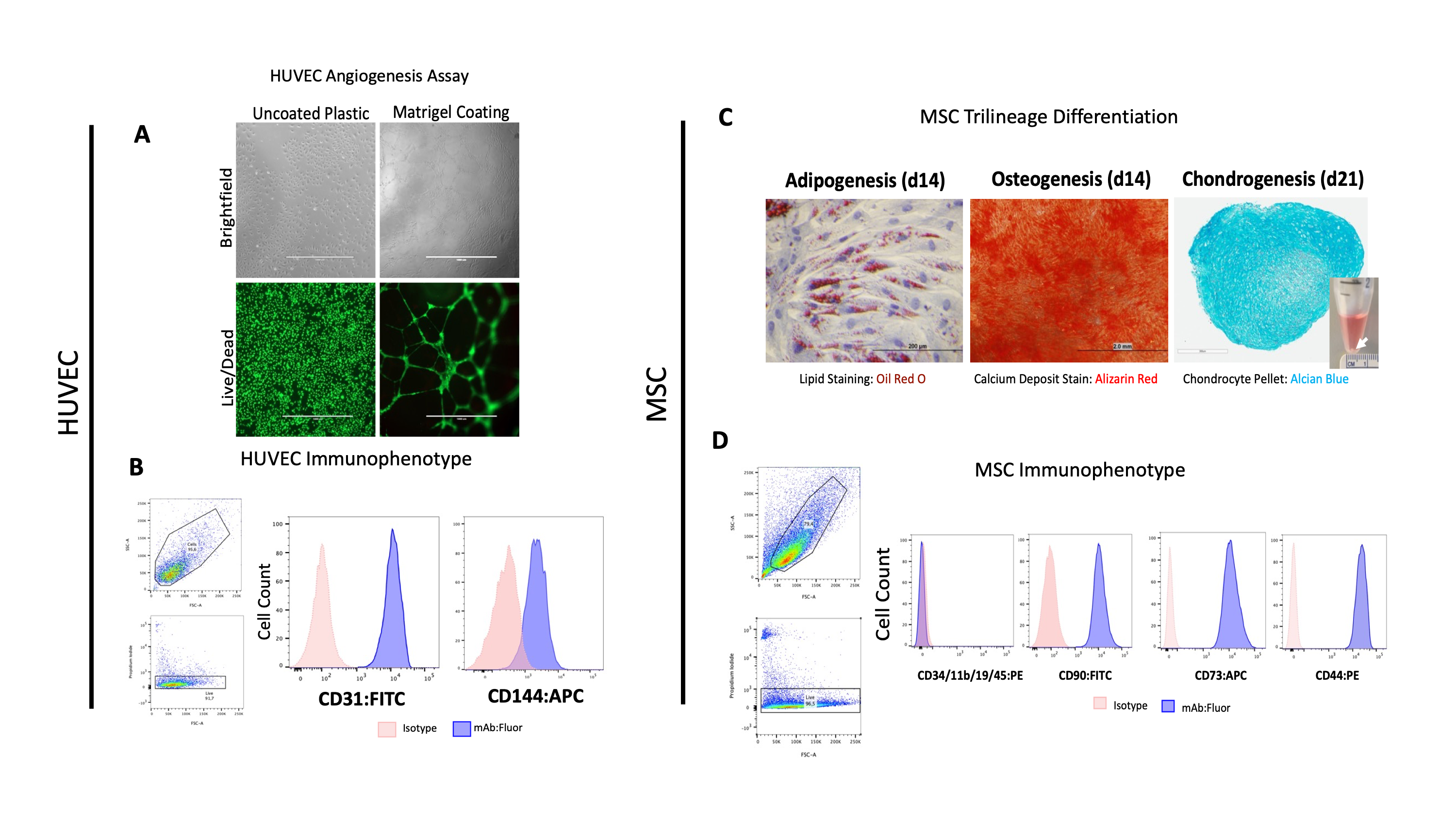

Supplement: Supplementary file 1 — Supplementary Figure 1. [file 41598_2024_58095_MOESM1_ESM.tiff]
