# Supplementary material for: Bioengineering of vascularized porcine flaps using perfusion-recellularization
Source: Sci Rep. 2024 Mar 31;14:7590. doi: 10.1038/s41598-024-58095-7 (PMC10981729; doi:10.1038/s41598-024-58095-7)
Supplement: Supplementary file 2 — Supplementary Information. [file 41598_2024_58095_MOESM2_ESM.docx]

**Supplemental Methods**

*Flow Cytometry*

In order to confirm endothelial cell phenotype, commercially obtained HUVEC (ATCC) at passage 5 were dissociated with trypsin-EDTA (Gibco) and single cell suspension was stained at a concentration of 1 x 10^5^/mL with monoclonal antibodies against CD31-FITC (BD Biosciences, USA), and VE-Cadherin-APC (Novus Biologicals, USA) after 1 hour incubation in the dark at room temperature.

For MSC phenotyping, commercially obtained bone-marrow derived MSC (Promocell) at passage 5 were analyzed using the BD Stemflow Human MSC Analysis Kit (BD Biosciences). Cells were stained with the included positive phenotype antibody cocktail containing CD90-FITC, CD44-PE and CD73-APC fluorochromes in addition to negative phenotype antibody cocktail containing CD45-PE, CD34-PE, CD11b-PE, CD19-PE and HLA-DR-PE fluorochromes after 1 hour incubation in the dark at room temperature.

After incubation with staining antibody, all cells were washed twice with PBS + 0.1% BSA. Conjugated isotype (IgG) antibodies were used as in all flow cytometry experiments as negative controls. Stained cells were analyzed with a flow cytometer (LSR-II Analyzer, BD Biosciences) with FACSDiva analysis software package. Each analysis were repeated in triplicated and included 100,000 events, after selection for viability and exclusion of debris.

*In Vitro Tube Angiogenesis Assay*

HUVECs were grown to 70-80% confluence in complete EGM 2 Media (Lonza) and dissociated with Trypsin-EDTA (Gibco). The single cell suspension was plated onto Matrigel (10mg/mL concentration; Corning) coated 96-well plates with final cell count of 25,000 cells per well to induce tube angiogenesis formation. Uncoated wells served as negative control for tube angiogenesis. Cells were incubated at 37°C/ 5 % CO_2_ for 20 hours prior to washing with PBS and staining with Calcein AM (Invitrogen) Live dye. Wells were imaged on standard epifluorescence microscope (EVOS FL, Invitrogen) with standard FITC filter.

*In Vitro Differentiation of MSC*

Commercially available MSCs (Promocell) were seeded at 3 x 10^4^ cells/cm^2^ in complete MSC Growth Media containing 5% FBS and were allowed to expand and reach approximately 100% confluence. Culture medium was then replaced with specific differentiation inductive medium. MSC Growth Media served as negative differentiation control. Culture media was changed every 2-3 days. For adipogenic differentiation, cells were cultured in MSC Adipogenic Differentiation Media 2 (Promocell) for 2 weeks. Differentiated MSC-derived adipocyte cell cultures were stained with Oil Red (Sigma Aldrich). For osteogenic differentiation, cells were cultured in the MSC Osteogenic Differentiation Kit (Promocell) for 2 weeks. Differentiated cultures were stained with Alizarin Red (Sigma Aldrich). For chondrocyte differentiation, MSCs were cultured in the MSC Chondrogenic Differentiation Kit (StemCell Technologies, Canada) at 1 x 10^6^ cells suspended within a 15mL conical tube to induce chondrogenic pellet formation. After 2 week incubation, the chondrocyte pellet was fixed in 10 % formalin (Fisher Scientific) and processed by routine histology and sectioned on microtome (Leica Biosystems) at 5µm thickness and stained with Alcian Blue (Sigma Aldrich) according to manufacturer instructions. All differentiation experiments were conducted with n = 3 replicates.

**Supplemental Figures**

**Supplemental Figure 1. In Vitro Phenotype and Functional Characterization of HUVEC and MSCs.** (A) HUVECs on Matrigel coated plasticware exhibit angiogenesis tube formation compared to culture on uncoated plastic. (B) Immunophenotype of HUVECs demonstrate positive cell surface markers CD31 (PECAM-1) and CD144 (VE-Cadherin). (C) Trilineage differentiation of MSCs into adipocytes, osteocytes, and chondrocytes were achieve after 14-day culture in appropriate differentiation media. Control media for adipogenesis and osteogenesis was standard MSC Growth Media (Promocell) containing 5% FBS. Note the formation of small cartilage pellet (white arrow) when MSCs are cultured in chondrogenic differentiation media. (D). Immunophenotype of MSCs demonstrate positive cell surface markers CD90, CD73, and CD44 as well as negative markers CD34, CD11b, CD19, and CD45. Representative microscopy images shown from n = 3 replicates. Scale Bars: 200 µm. Both HUVEC and MSCs were used at passage 5 for all flow cytometry and functional assay experiments.
